# Honey bee genetics shape the strain-level structure of gut microbiota in social transmission

**DOI:** 10.1186/s40168-021-01174-y

**Published:** 2021-11-17

**Authors:** Jiaqiang Wu, Haoyu Lang, Xiaohuan Mu, Zijing Zhang, Qinzhi Su, Xiaosong Hu, Hao Zheng

**Affiliations:** College of Food Science and Nutritional Engineering, China Agricultural University, Beijing, 100083 China

**Keywords:** *Apis mellifera*, Gut microbiota, GWAS, *Bifidobacterium*, Host specificity, Type IV pili, Polysaccharide utilization loci

## Abstract

**Background:**

Honey bee gut microbiota transmitted via social interactions are beneficial to the host health. Although the microbial community is relatively stable, individual variations and high strain-level diversity have been detected across honey bees. Although the bee gut microbiota structure is influenced by environmental factors, the heritability of the gut members and the contribution of the host genetics remains elusive. Considering bees within a colony are not readily genetically identical due to the polyandry of the queen, we hypothesize that the microbiota structure can be shaped by host genetics.

**Results:**

We used shotgun metagenomics to simultaneously profile the microbiota and host genotypes of bees from hives of four different subspecies. Gut composition is more distant between genetically different bees at both phylotype- and “sequence-discrete population” levels. We then performed a successive passaging experiment within colonies of hybrid bees generated by artificial insemination, which revealed that the microbial composition dramatically shifts across batches of bees during the social transmission. Specifically, different strains from the phylotype of *Snodgrassella alvi* are preferentially selected by genetically varied hosts, and strains from different hosts show a remarkably biased distribution of single-nucleotide polymorphism in the Type IV pili loci. Genome-wide association analysis identified that the relative abundance of a cluster of *Bifidobacterium* strains is associated with the host glutamate receptor gene specifically expressed in the bee brain. Finally, mono-colonization of *Bifidobacterium* with a specific polysaccharide utilization locus impacts the alternative splicing of the *gluR-B* gene, which is associated with an increased GABA level in the brain.

**Conclusions:**

Our results indicated that host genetics influence the bee gut composition and suggest a gut-brain connection implicated in the gut bacterial strain preference. Honey bees have been used extensively as a model organism for social behaviors, genetics, and the gut microbiome. Further identification of host genetic function as a shaping force of microbial structure will advance our understanding of the host-microbe interactions.

**Video abstract**

**Supplementary Information:**

The online version contains supplementary material available at 10.1186/s40168-021-01174-y.

## Background

It is becoming increasingly clear that most animals are universally inhabited by microbial communities in their guts. These host-associated microbiomes provide considerable benefits to the host through different functions and shape the host’s ecology and evolution [1]. Microbiomes in animals are often acquired at birth and contact with others and the environment. Considering the importance of the microbiome, it is thus crucial to understand mechanisms that select, retain, and transfer the commensal microbes and the impact of specific constituents on host biology. Numerous environmental factors, including lifestyle, diet, disease, geography, and medications, influence the gut microbiota [2–4]. In addition to the external factors, host genetics has recently been proposed as a determinant of gut microbial composition [5]. Studies searched for associations between host alleles and gut microbiota structure of humans, mice, and other animal models [6–8]. Different genetic loci have emerged as influential in accounting for interindividual variation in microbiomes; specifically, genes with roles in host metabolism and immune systems implicated in disease have been studied for their impact on the microbiome [9–12]. In addition, a suite of taxa is considered more heritable than others, suggesting that host genetic variation can explain the levels of specific gut members. Particular taxa, such as Christensenellaceae and methanogens, are linked to the host lean phenotype, suggesting the important functions and underlying host-microbe interactions of these highly heritable gut members [13–15]. The potential interactions of host genes and the microbiome have been surveyed for humans and other animals. Yet, it is challenging for the association of specific host alleles with microbiome traits [7]. This is partly due to the gut community structure being strongly and rapidly affected by various environmental factors, making it challenging to compare animals in controlled laboratory settings, limiting our ability to extrapolate host-microbe interactions. Moreover, the genome-wide association study (GWAS) for hosts with a relatively large genome size with complex microbiota dominated by thousands of bacterial species is costly [16]. Therefore, experimental systems with a simple microbiota composition that can be controlled in designated conditions are required to better understand the processes that determine microbiome structure and transmission dynamics.

Honey bees possess a simple and host-restricted gut microbiota commonly detected in bees that are even derived from different locations [17, 18]. This community contains only 5–8 core bacterial phylotypes (with > 97% sequence similarity in the 16S rRNA gene), whereas multiple “sequence-discrete populations” (SDPs; with ~ 90% genomic average nucleotide identity) and strains have been defined in each phylotype, indicating a strain-level variation in the bee gut [19, 20]. In particular, although only one phylotype has been identified for the core gut member *Snodgrassella*, high strain-level diversity and host-specific pattern have been indicated [21, 22]. In addition, multiple strains from all core members of bee gut bacteria have been isolated, and the ease of rearing microbiota-free (MF) bees facilitates a functional screen of individual gut members using gnotobiotic bees inoculated with defined communities [23]. Thus, the bee gut community provides an excellent model for studies on the processes that govern the assembly of microbiota. To date, studies have focused on the roles of bee gut microbiota, such as their effects on host nutrition, weight gain, endocrine signaling, and immune functions [24, 25]. However, the knowledge of the effectors that affect the composition and transmission of the honey bee gut microbiota is quite limiting.

It has been documented that the characteristic bee gut microbiota is mainly acquired from the other nestmates through social contact, and the bee gut microbiota are vertically inherited inside the colony [26]. Although the western honey bee (*Apis mellifera*) gut microbiota are relatively stable across colonies from different geographic locations, individuals with varying host behaviors and physiologies, such as age and caste, possess distinctive gut compositions [27, 28]. Moreover, gut compositions vary in individuals from the same colony, with high diversity in strain composition for the core gut members observed in particular [21]. Worker bees from the same hive are not readily genetically identical since queen bees are polyandrous and mate with an average of 12 males to produce daughters of mixed paternity [29, 30]. Indeed, a previous study showed that colonies with genetically diverse populations exhibit a higher microbiota diversity, strongly suggesting a host genetic impact on the gut community [31]. Therefore, we hypothesized that host genetics is a shaping force of the bee gut microbial diversity, especially the strain-level variation and the dynamics of microbiota transmission along with generations.

Here, using shotgun metagenomics, we simultaneously profiled the strain-level bacterial community and host genotype for a pure race of bees and hybrids generated through artificial insemination. It was observed that the abundance of most core gut members was influenced by host genetics. A longitudinal study of the structure and dynamics of the gut communities found that specific taxa were selected by the host during the successive passage across genetically varied generations. Heritability analysis and a GWAS on gut composition identified a significant association between *Bifidobacterium* and the G-protein-coupled receptor gene preferably expressed in the bee brain. Furthermore, the brains of bees colonized with the heritable *Bifidobacterium* strain displayed an increased level of GABA and differential splicing events of the *gluR-B* gene in the brain, which may be explained by a glycan utilization gene cluster specific to the highly heritable strains.

## Methods

### Honey bee management and samples collection

All honey bees (*Apis mellifera*) were bred and maintained in the apiary of Jilin Province Institute of Apicultural Science. The purebred swarms were established in the 1980s when the queens were imported, and all breeds were conserved by artificial insemination each year for long-term genetic studies. Four different subspecies, namely OH, AF, YF, and SK, were used in this study. We set up one hive for OH and AF each and two hives of YF and SK each, and both the purebred and hybrid colonies were constructed in July 2018. For the hybrid colonies, virgin queens of OH and drones of AF or YF were mated by artificial insemination to produce hybrids O-A and O-Y, respectively. The queens were singly mated by being instrumentally inseminated with semen harvested from a single drone following the protocols described in COLOSS BeeBook [32]. Then the inseminated queens were placed into nucleus colonies with ~ 300 founding workers, named O-A’ and O-Y’ here. No aggressive behavior of the founding workers was observed against the newly-inseminated queens nor the newly emerged hybrids. When the queens started laying eggs, each colony was monitored daily. To control the age of sampled bees, one-day-old workers were obtained by moving frames containing late-stage pupae from colonies into an incubator (35 °C, 50% relative humidity) overnight. The newly emerged adults in the incubator were individually marked on their thoraces with a spot of color paint and were then placed back into their original hives. All individuals were collected when they are 15 days old. When the first batch of bees (B1) were collected, the second batch (B2) was set up as described above. Three batches of bees (B1–B3) were labeled with distinct colors and sampled consecutively. Notably, before the emergence of B3, it had been more than 50 days since the founding workers were introduced. Thus, all O-A’ and O-Y’ bees had died. In total, we sampled 335 individual bees for the microbiome structure analysis, using either shotgun metagenomics or 16S rRNA amplicon sequencing. Details of the sample size are listed in Table S1 (Additional file : Table S1).

Colony development of the hybrid colonies was recorded at the end of the experiment as described in the COLOSS BeeBook [33]. Briefly, the number of adult workers was estimated by weighing the net colony bee weight. The whole hive is weighed in the field, and then all bees were brushed off each comb into a holding container. The hives were re-weighed without bees. We then collected ~ 300 bee individuals into a pre-weighed container. After immobilization at 4 °C for 30 min, the bees were counted, and the average fresh weight was determined. Then, net colony bee weight is divided by the average individual weight to estimate the population size.

### Generation of mono-colonized honey bees

Mono-colonized bees were obtained as described by Zheng et al. [34] with modifications*.* Late-stage pupae were removed manually from brood frames and placed in sterile plastic bins. The pupae emerged in an incubator at 35 °C, with a humidity of 50%. Newly emerged MF bees were kept in axenic cup cages (20–25 MF bees per cup cage) with sterilized sucrose syrup (50%, wt/vol) for 24 h. For the mono-colonization groups, stocks of *Bifidobacterium asteroides* strain W8113 and strain W8111 in 25% glycerol stock at − 80 °C were resuspended in 1mL 1×PBS (Solarbio, Beijing, China) at a final OD_600nm_ of 1. Bacterial cell suspensions were mixed with 1 ml sterilized sucrose solution (50%, wt/vol) and 0.3 g sterilized pollen. After a 24-h feeding, mono-colonized bees were provided with sterilized sucrose (0.5 M) and sterile pollens and were kept in an incubator (35 °C, RH 50%) until day 7.

### DNA extraction and shotgun sequencing

All bee individuals of either purebred or hybrid were sampled exactly 15 days after the emergence. Total genomic DNA of both the bee host and gut microbiota was extracted from the whole gut homogenate using the CTAB method as previously described [24]. DNA samples were sent to Guangdong Magigene Biotechnology (Guangzhou, China) for shotgun metagenome sequencing. Sequencing libraries were generated using NEBNext UltraTM II DNA Library Prep Kit for Illumina (New England Biolabs, MA, USA), and the library quality was assessed on Qubit 3.0 Fluorometer (Life Technologies, Grand Island, NY) and Agilent 4200 (Agilent, Santa Clara, CA) system. The libraries were then sequenced on the Illumina HiSeq X-ten platform with 150-bp paired-end reads.

### Mapping and variant calling on honey bee genomes

The raw data obtained from the Illumina HiSeq sequencing platform was preprocessed by Readfq: reads with low-quality bases above 40 bp (quality value ≤ 38), with N bases > 10 bp were removed, and sharing the overlap > 15 bp with the adapters were all removed. In addition, reads were quality controlled with Fastp using default parameters to generate clean data for downstream analysis. ~ 10 Gb of sequences per sample were obtained for subsequent analyses. The quality-controlled reads were mapped to the honey bee reference genome (version Amel_HAv3.1; GenBank assembly accession: GCA_003254395.2) using the BWA-MEM algorithm with the option “-t 4, -R, -M.” We then sorted the alignments according to mapping coordinates and marked PCR duplicates using Picard Tools, and the duplications were removed using the SAMtools program to create one BAM file for each bee sample. To improve mapping quality, we set minimum mapping quality = 1. We defined depth of coverage as the number of mapped bases divided by the total length of the reference genome, and the coverage of more than 90% of the samples is above 20×. As a primary quality control metric, 95–100% (median 99.14%) of mapped reads per sample were properly in pairs.

We called variants using the Genome Analysis Toolkit v4.0.3.0 following germline short variant discovery pipeline of the best practices. In brief, the HaplotypeCaller module with default parameters was used to calculate variant calls independently for each BAM file and generate one gVCF file per sample. Then, gVCFs were merged into GenimicsDB across all samples by GenomicsDBImport module for the following joint genotyping, which was performed using GenotypeGVCF on all samples to create a single VCF file for the whole population. To improve the quality of the variants, we filtered out false-positive SNPs with VCFtools and checked the dataset by VariantQC. Finally, only sites with a minor allele frequency between 0.05 and 0.95 were kept. The quality of the final set of SNPs was assessed by calculating the ratios of transition to transversion (Ti/Tv ratio), a diagnostic parameter to examine the quality of SNP identification. The ratios were between 4.17 and 4.29 in our population, which indicates no excess of false-positive SNPs.

### Honey bee population genetics analysis

To investigate the genetic relatedness of purebred bees, including OH, YF, AF, and SK, the gVCF file of *Apis cerana* (version ACSNU-2.0) was merged with the population VCF dataset of *A. mellifera* created above. We then measured the raw genetic distance using the ‘SNPRelate’ package based on the number of shared alleles between each sample. The distance matrix was used to estimate a tree using the neighbor-joining method implemented in R package ‘ape’, and the tree was visualized using iTol. PLINK was used to generate a clear population VCF dataset with only biallelic loci; all variants with missing call rates exceeding 0.05 were filtered out. Markers with a Hardy-Weinberg Equilibrium *p*-value < 0.0001 and individuals with less than 0.05 missing genotype data were excluded. In total, we identified 2,255,909 high quality variants (average value = 20,482 per site) across the 57 purebred bees. The numbers of SNVs from all samples presented across *A. mellifera* chromosome 1 to 16 in 100-kb consecutive windows are shown in Fig. S2. We also processed the same pipeline to acquire variant files of hybrid and founding worker bees (Figure S4), which resulted in 2,444,291 variants (average value = 31,690 per site) across the 68 individuals. We then ran ADMIXTURE on the resulting SNPs to estimate the genetic ancestry of each sample. The unsupervised analysis was performed using hypothetical ancestral components (*K*) ranging from 2 to 5. The fivefold cross-validation (CV) error values were estimated in ADMIXTURE at each *K* value.

### Isolation and genome sequencing of gut bacteria

Bacterial strains were isolated from the guts of *A. mellifera* collected in Jilin, China, during July 2018. The dissected guts were directly crushed in 20% (vol/vol) glycerol and frozen at − 80 °C after sampling. The glycerol stocks were plated on heart infusion agar supplemented with 5% (vol/vol) defibrinated sheep’s blood (Solarbio, Beijing, China), MRS agar (Solarbio, Beijing, China) or TPY agar (Solarbio, Beijing, China) incubated at 35 °C under a CO_2_-enriched atmosphere (5%). Genomic DNA of bacterial isolates was extracted using the CTAB buffer method as previously described [34]. The colonies were first checked by Sanger sequencing of the16S rRNA. Then, distantly related isolates based on the phylogeny of 16S rRNA sequences within each SDP were chosen for genome sequencing. Total genomic DNA of the isolate was sequenced on the Illumina Nova6000 platform from the 150-bp paired-end libraries, and the quality-controlled reads were assembled with the SOAPdenovo2 genome assembler. The completeness of genomes was assessed by CheckM. Whole-genome average nucleotide identity (ANI) was calculated using FastANI. The genomes were annotated with the Prokka software, and phylogenetic trees were reconstructed based on the whole-genome sequence information by PhyloPhlAn. All genome assemblies obtained in this study were deposited at DDBJ/EMBL/GenBank, and the accession numbers are listed in Additional file : Dataset S1.

### Metagenomic analysis

The SDP- and strain-level community structure of each sample was profiled following the Metagenomic Intra-Species Diversity Analysis System (MIDAS) pipeline. Firstly, a custom genomic database for taxonomy classification and strain SNP calling was built using genomes of 116 bee gut bacterial isolates obtained in this study together with 289 published genomes isolated from *Apis* and *Bombus* for the downstream MIDAS analysis (Additional file : Dataset S1). Firstly, pairwise genomic average nucleotide identities (ANI) for strains within each phylotype were calculated with fastANI. According to the MIDAS pipeline instruction, genomes with an exact 95% ANI pairwise similarity were grouped into one species cluster, defined as “MIDAS-taxonomy” here. Then we built maximum-likelihood trees for the five core gut members using FastTree based on the amino acid sequences (Additional file : Figure S1). Sequence-discrete populations were basically defined as described by Ellegaard et al. [19]. SDPs have been recently proposed to represent bacterial species [35]. They are groups of divergent strains with less than 90% gANI and form separate phylogenetic clusters. Our analyses of the isolate genome phylogeny and pairwise genomic ANI confirmed the existence of most of the SPDs. However, we noticed that the pairwise ANI within the predefined SDP “Bifido-1” is much lower than the expected threshold (95%).

Moreover, our previous work has documented that strains from “Bifido-1” show dramatically various capacities in polysaccharide digestion [34], indicating that they might belong to different bacterial populations. Here, we included 28 new bifidobacteria isolates in the analyses of genome phylogeny and pairwise genomic ANI. We identified that “Bifido-1” could be further divided into four SDPs, which form discrete clades and show similar metabolic profiles [34]. Thus, the bacterial genomes were integrated into 76 MIDAS-species clusters, and they were further grouped into 17 SDPs for the five core phylotypes and *Bartonella apis*. There are also 20 SDPs for the other non-core members included in the database for the taxonomy classification. The taxonomic annotations of the MIDAS custom database are shown in Additional file : Dataset S1.

Before the taxonomic profiling, we removed host-derived reads from metagenomic clean data with KneadData, which maps the reads to the honey bee reference genome (Amel_HAv3.1) with default parameters and performs quality control. To compute the relative abundance of SDPs, coverage, and prevalence from metagenomic sequencing, we ran the “species” module of the “run_midas.py” script and “merge_midas.py” script with our custom bacterial genome database. MIDAS aligned reads to the database of universal single-copy marker gene sequences for each species cluster within our custom database using HS-BLASTN to estimate the abundance of species clusters for each sample. Local alignments that cover < 70% of the read or fail to satisfy the gene-specific species-level percent identity cutoffs were discarded, and we assigned each uniquely mapped read to a species cluster according to its best hit. Then, each species cluster's coverage and relative abundance across samples were estimated based on the number of mapped reads. We compared both phylotype- and SDP-level composition profiles by measuring Bray-Curtis distance using the “vegan” package. First, we used Wilcoxon tests to evaluate the dissimilarity for each subject. Then, we compared the relative abundance of all phylotypes and SDPs for different subspecies of bees. We used the Kruskal-Wallis test to determine the significance.

### Strain-level community profiling

Representative genomes from each species cluster were chosen to maximize its average nucleotide identity at the 30 universal genes to other members of the species cluster, and they were used for detecting core-genome SNPs. Next, a database of 15 universal single-copy gene families was built to estimate the abundance of the species clusters from the shotgun metagenomes. Universal and single-copy gene families were selected by MIDAS based on their ability to recruit metagenomic reads accurately. We used the “single-nucleotide-polymorphism prediction” function of the MIDAS pipeline to profile SNPs of the five core SDPs in metagenomic data for each bee against the representative genomes. In brief, we ran the “snps” module of the “run_midas.py” script to map metagenomic reads to the reference genomes. Representative genomes with the highest completeness from each SDP were selected for the strain SNP calling. Metagenomic reads were aligned to the reference genomes using Bowtie2, with default MIDAS mapping thresholds: global alignment, mapping percent identity ≥ 94.0%, sequence quality ≥ 20, alignment coverage ≥ 0.75, and mapping quality ≥ 20. Pileups of each sample were generated using SAMtools, and the nucleotide variation statistics were then counted at each genomic site. After running this script for each sample, we pooled all samples. Next, we performed core-genome SNP calling using the “snps” module of the “merge_midas.py” script. The core-genome SNP matrices were produced to compare nucleotide variation across genomic sites and metagenomic samples of different bee groups. Specifically, bi-alleles genomic sites present in more than 95% of strains and with a minimum prevalence frequency of 1% were identified as core SNPs using the “--core-snps” parameter. MIDAS reported the read coverage for each site in the reference genome for each metagenomic sample based on the raw alignments. The frequencies of both silent and missense allele per genomic site per sample in protein-coding genes were used to indicate the strain-level variation among different individuals as previously described [36, 37].

### 16S rRNA high throughput sequencing

The V4 region of the 16S rRNA gene was amplified (primers 515F and 806R) with barcodes. All PCR reactions were carried out with 15 μL of Phusion® High-Fidelity PCR Master Mix (New England Biolabs); 0.2 μM each of forward and reverse primers, and 10 ng template DNA. PCR products were purified with Qiagen Gel Extraction Kit (Qiagen, Germany). Sequencing libraries were generated using NEBNext® UltraTM II DNA Library Prep Kit for Illumina® (New England Biolabs, MA, USA), and index codes were added. The library quality was assessed on the Qubit 2.0 Fluorometer (Thermo Fisher Scientific, MA, USA). The library was sequenced on an Illumina Nova6000 platform with 250-bp paired-end reads, and at least 30,000 sequences were obtained for each sample. Fastp [38] was used to control the raw data quality by sliding window (window size = 4 bp, mean quality = 20). The primers were removed using Cutadapt software [39] according to the primer information at the beginning and end of sequences to obtain clean reads. Mate-pair merging, OTU picking, chimera elimination, and taxonomy classification were performed using Mothur version 1.40.5 following the MiSeq standard operating procedure [40]. 16S rRNA gene sequences were clustered into Operational Taxonomic Units (OTUs) at a similarity cutoff value of 99%. We used a curated database for bee gut microbiota based on the SILVA database for the classification [41]. The subsequent analysis was based on a normalized OTU table using the “phyloseq” package [42], and Bray-Curtis diversity was calculated with the “vegan” package [43].

### Heritability calculations

The heritability of each gut bacteria taxa is defined as the proportion of total variance due to genetic effects as previously described [11]. The heritability was calculated using the additive effect model in HIBLUP V1.3.1 under the Genomic Best Linear Unbiased Prediction framework (https://hiblup.github.io). The VCF datasets of OH, YF, AF, SK, O-A, and O-Y groups were used here. Variants in the host genome were quality controlled; sites with a minor allele frequency < 0.05 or > 0.95 or failed in the Hardy-Weinberg test at 0.0001 were removed, which results in 2,861,994 informative SNPs across 102 bee samples, and the average quality value is 49,095. A combination of the HE Regression and Average Information algorithms was used to obtain an efficient and robust variance component estimation. Grouping of hives and kinship among individuals are used as random effects.

### Bacterial associations to host genetic variation

We performed GWAS analysis for the relative abundances of each core bacteria phylotype and SDP using rMVP v1.0.0 with correcting for population structure. We used the SNP profiles of 102 individuals from OH, YF, AF, SK, O-A, and O-Y groups of bees from the clear VCF dataset. All sites were filtered with PLINK software as described above. General Linear Model (GLM) and Mixed Linear Model (MLM) were used for the host SNP-microbe association tests, and we used the ‘GEMMA’ method to analyze the variance components. The relatedness matrix, measured as the genetic similarity between individual bees, was used to estimate random effects. For all samples, SNPs and the top three PCs were used as fixed effects in MLM, and the top five PCs were used in GLM. The p-values were firstly set at 0.05 for each association test. Then the Benjamini-Hochberg corrected p-value threshold for all SNPs was used to control false-positive error rates deriving from multiple testing at the genome-wide level.

### Targeted metabolomics for GABA in bee brains

Brain tissues of individual bees were collected using a dissecting microscope (Canon). Individual bee was fixed on beeswax using two insect needles through the thorax. After removing the head cuticle, the whole brain was taken out on the glass slide, placed on top of an ice pack. The hypopharyngeal glands, salivary glands, three simple eyes, and two compound eyes were carefully removed. Brain tissues dissected from mono-colonized bees were sent to Biotree Biotech Co. Ltd. (Shanghai, China) for targeted metabolomics analysis of GABA. Six brain tissues from one treatment group were put into one tube and centrifuged (2400 g × 1 min at 4 °C). A total of 100 μL of acetonitrile containing 0.1% formic acid and 20 μL of ultrapure water were added, and the tubes were vortexed thoroughly. Tissue cells were sonicated in an ice-water bath for 30 min, followed by subsiding at − 20 °C for 2 h. Supernatants were collected after centrifugation (14,000 g × 10 min at 4 °C). Next, 20 μL of the supernatant was transferred to a new vial followed by incubation for 30 min after adding 10 μL sodium carbonate solution (100 mM) and 10 μL 2% benzoyl chloride acetonitrile. Then, 1.6 μL internal standard and 20 μL 0.1% formic acid were added, and the samples were centrifuged (14,000 g × 5 min at 4 °C). A total of 40 μL of the supernatants were transferred to an auto-sampler vial for downstream UHPLC-MS/MS analysis. 4-aminobutyric acid (Sigma-Aldrich) was used for the construction of the calibration standard curve.

The UHPLC separation was carried out using an ExionLC System (AB SCIEX; MA, USA), and the samples were analyzed on the QTRAP 6500 LC-MS/MS system (AB Sciex; Framingham, MA, USA). Two microliters of samples were directly injected onto an ACQUITY UPLC HSS T3 column (100 × 2.1 mm × 1.8 μm; Waters; Milford, Ma, USA). The column temperature was set at 40 °C, and the auto-sampler temperature was set at 4 °C. Chromatographic separation was achieved using a 0.30 ml/min flow rate and a linear gradient of 0 to 2% B within 2 min; 2–98% B in 9 min, followed by 98% B for 2 min and equilibration for 2 min. Solvent A is 0.1% formic acid, and solvent B is acetonitrile. For all multiple reaction monitoring (MRM) experiments, 6500 QTrap acquisition parameters were as follows: 5000 V Ion-spray voltage, curtain gas setting of 35, and nebulizer gas setting of 60, the temperature at 400 °C. Raw data were analyzed using Skyline [44].

### RNA extraction and brain gene expression

Dissected brains were kept frozen at – 80 °C in RNAlater (Thermo Fischer; Waltham, MA, USA). Total RNA was extracted from individual brains using the Quick-RNA MiniPrep kit (Zymo Research). RNA degradation and contamination were monitored on 1% agarose gels, and the purity was checked with the NanoPhotometer spectrophotometer (IMPLEN; CA, USA). RNA integrity was assessed using the RNA Nano 6000 Assay Kit of the Bioanalyzer 2100 system (Agilent Technologies, CA, USA).

RNA sequencing libraries were generated using NEBNext Ultra RNA Library Prep Kit for Illumina (New England BioLabs; Ipswich, MA, USA). Index codes were added to attribute sequences to each sample. The clustering of the index-coded samples was performed on a cBot Cluster Generation System using TruSeq PE Cluster Kit v3-cBot-HS (Illumina). The library preparations were then sequenced on an Illumina NovaSeq 6000 platform, and 150-bp paired-end reads were generated. The sequencing quality of individual samples was assessed using FastQC v0.11.5 with default parameters. An index of the bee reference genome (Amel_HAv3.1) was built using HISAT2 v2.0.5 [45], and the FastQC trimmed reads were then aligned to the created index using HISAT2 v2.1.0 with default parameters. StringTie v1.3.3 [46] was then applied to assemble the obtained alignments in a BAM format into potential transcripts, using the GTF file of the honey bee genome downloaded from NCBI as a reference. Then we merged transcripts from all samples and examine how the merged transcripts compare with the reference annotation. The gene and transcript abundances were estimated using merged transcripts as reference.

Before differential gene expression, we first transformed the abundance into raw counts using scripts offered by StringTie. Count-level data underwent relative log expression (RLE) to estimate size factor and dispersion, then fit for each gene with fitType “local,” followed by Wald test to determine differential gene expression (DGE) between bees mono-colonized with *Bifidobacterium asteroides* strain W8113 and W8111 from “Bifido-1.4” using the R package DESeq2 v1.22.2 [47]. Analysis of event-level differential splicing was performed using rMATS v4.0.2 [48] based on the newly merged transcripts in StringTie as reference. An exon-based ratio metric, commonly defined as percent-spliced-in value, was employed to measure the alternative splicing events. The percent spliced in (PSI) value is calculated as follows:

$$\varphi =\frac{\frac{I}{l_I}}{\frac{I}{l_I}+\frac{S}{l_S}}$$

where *S* and *I* are the numbers of reads mapped to the junction supporting skipping and inclusion form, respectively. Effective length *l* is used for normalization. The PSI value was calculated for different classes of alternative splicing events, including skipped exon (SE), alternative 5′ splice site (A5SS), alternative 3′ splice site (A3SS), mutually exclusive exons (MXE), and retained introns (RI). Events with *p* < 0.05 were considered differentially spliced between the two groups of bees.

### Statistical analysis

Wilcoxon test was used to determine the significance of Bray-Curtis distance or the relative abundance of each microbiota taxon between each two bee groups, and Kruskal-Wallis test was used for multi groups. The threshold for genome-wide significance was corrected for multiple testing with a weighted Bonferroni adjustment, with adjusted *p* < 0.05 as significant. GLM and MLM are implemented for association tests.

## Results

### Gut communities are more different between genetically varied honey bees

**Fig. 1:**
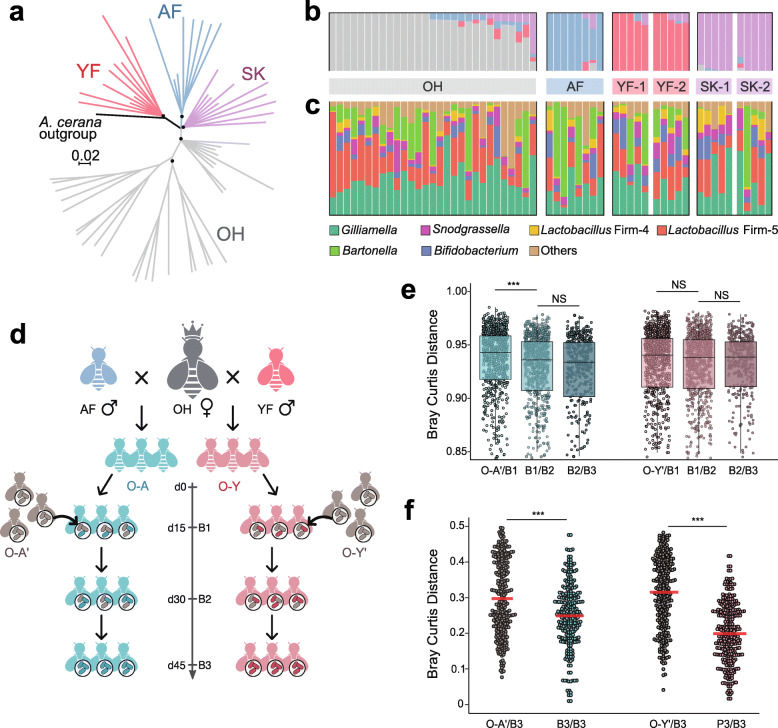
Gut microbiota compositions differ across genetically varied honey bees. **a** Neighbor-joining tree constructed from allele-sharing distances between honey bee subspecies. Nodes with 100% support are marked with dots. The scale bar represents raw genetic distance per variable site. **b** ADMIXTURE analysis showing clustering of bee samples into four groups (*K* = 4). One colony each of OH (*n* = 29) and AF bees (*n* = 8), and two colonies each of YF (*n* = 10) and SK (*n* = 10) bees were sampled for the metagenomic analysis. Each bar represents one individual bee. **c** Relative abundance of phylotypes in the guts of bee individuals from different subspecies. **d** Study design for serial transmission of gut microbiota in two lines of hybrid bees generated by artificial insemination. We sampled guts from three batches of hybrid bees (B1–B3) during the passage and from the founding workers that initiate the colony (O-A’, O-Y’). **e** Bray-Curtis distance of the gut communities between bees from different batches at the OTU-level of 16S rRNA sequences. **f** Bray-Curtis distance of the gut communities at the SDP level by metagenomic sequencing. Wilcoxon test was used to compare the average of Bray-Curtis distance between each two bee groups, and a *p* value < 0.05 indicates statistical significance. (NS, not significant; ***p < 0.001)

*A. mellifera* belonging to four different subspecies, namely OH, AF, YF, and SK, were sampled. OH is one subspecies of the European dark bee, and the others are yellow bee species. They were imported into China in the 1980s and were then kept in Jilin province for germplasm conservation. Metagenomic sequencing of gut homogenates from 57 individual bees from the four subspecies was performed, and bees from YK and SK were sampled from two independent hives each. We processed the whole gut for shotgun sequencing, thus simultaneously acquiring the genomic information of the host and microbial community. For each sample, 53–127 million pair-end reads (150 bp) were generated. First, to determine the genomic diversity of the hosts, the sequencing reads were mapped to the honey bee genome assembly (version Amel_HAv3.1). A total of 33–77% of the reads were mapped to the honey bee genome, indicating a 13–57× coverage of the honey bee genome, and 2,255,909 sites were identified as polymorphic (Additional file : Figure S2a). An evolutionary tree of *A. mellifera* inferred from all single-nucleotide polymorphism (SNP) demonstrated apparent clustering of the four groups (Fig. 1a). The OH bees were more distantly related to the other three subspecies. Likewise, ADMIXTURE analysis of genetic co-ancestry also partitioned the data population into the four defined groups when the number of populations was set at *K* = 4 (Fig. 1b, Additional file : Figure S2b). Different subspecies had an average pairwise *F*_ST_ (allelic fixation index) of 0.08–0.11, which was consistent with a previous analysis of *A. mellifera* subspecies [49]. These results supported the designation of subspecies with distinct genetic backgrounds.

We then assessed the composition of the gut community using the MIDAS pipeline with a custom database [36]. To construct a better reference database for the analyses of strain-level genomic variation, we isolated 116 bacterial strains from the gut homogenates of *A. mellifera*, and a new genomic database was generated using 405 bacterial genomes from both honey and bumble bee gut isolates (see Methods). For all samples, an average of 26% of reads was mapped to the bacterial database. The relative abundances of bacterial phylotypes and SDP were estimated using MIDAS, which maps reads to a panel of 15 selected marker genes of the genomes. Although many reads were mapped to the host genome, the accumulative curves of the observed SDPs began to plateau, indicating that the microbial dataset was adequate for the diversity analyses (Additional file : Figure S3). Consistent with previous 16S rRNA- and metagenome-based studies [19, 50], all individual gut communities were dominated by the five core bacterial species. *Gilliamella* and *Lactobacillus* Firm5 were the most abundant members, whereas some AF and OH bees had a higher fraction of *Bartonella* (Fig. 1c). Although it was suggested that wintering bees possess more *Bartonella* [51], this study was performed in July 2018. All individuals were sampled on exactly Day 15 following the emergence, suggesting it is not a seasonal or age effect. In addition to the core members, the other non-core species, including *Frischella perrara*, *Lactobacillus kunkeei*, and *Commensalibacter* spp., were detected in variable amounts. Since the honey bee gut community had a low complexity at the phylotype level, the SDP-level profiles of different bee subspecies were then compared. First, all SDPs from each phylotype co-occurred in individual bees. While the relative abundance of different phylotypes was not different, the SDP-level profiles were more variable between individuals with different genetic backgrounds. For example, although the frequency of *Bifidobacterium* was not different among subspecies of bees, OH and AF had more strains from the Bifido-1.2 SDP, while Bifido-1.4 was more abundant in YF and SK bees (Additional file : Figure S2c). For *Gilliamella*, Gilli-1 was the predominant SDP, while Gilli-3 was only present in tiny amounts in all bees, and the frequencies were not associated with host genetics. In summary, our results showed that the gut community compositions of bees with a varied genetic background were different, suggesting that the host genetic variation is associated with the gut microbiota profiles.

### Host genotype determines the composition of the passaged gut community

The gut microbiota of honey bees are acquired via social interactions with other workers but not through a maternal vertical transmission [52]. Thus, the gut microbiota is transmitted in the colony between batches of siblings from the same queen. To determine if the host genotype impacts the socially transmitted microbiota, we started two lines of colonies (three replicate hives each) headed by OH virgin queens instrumentally inseminated with semen from single drones of AF or YF (Fig. 1d). Then colonies were initiated by the inseminated queens, together with ~300 founding workers (O-A’ and O-Y’) randomly sampled from one hive without control of host genetic background, who fed the hybrids at the beginning (O-A and O-Y). Thus, the gut microbiota of the hybrids must have been derived from the founding workers and then transmitted among batches of hybrids within the colonies. Three different batches of newly emerged hybrid adults were marked with color paints, and they were sampled when they were exactly 15 days old. It is noteworthy that, before the third batch of bees started to emerge, all initial founding workers had died. At the end of the experiment (~ 66 days after colony initiation), the population size of both O-A and O-Y colonies and the fresh weight of individual bees were checked. There were no significant hive variations, so the genetic background did not alter the colony’s growth (Additional file : Figure S4a, b).

ADMIXTURE analysis showed that the genetic backgrounds of the founding workers were genetically different from the hybrid bees (Additional file : Figure S4c). Next, the impact of host genotypes on bacterial community transmission was measured by 16S rRNA sequencing of the gut microbiota of the founding bees, together with the three batches of hybrid workers (B1–B3). By testing OTU-level Bray-Curtis dissimilarity between adjacent time points for each batch of individuals, it was found that gut communities of hybrids with more similar genetic backgrounds (B1/B2 and B2/B3) exhibited similar features over time (Fig. 1e). However, gut communities of B1 markedly changed from those of the founding bees of O-A’. While the gut community did not shift between O-Y and O-Y’ bees at the phylotype level, we next determine the fractions of all SDPs in the founding bees and the B3 batch (Additional file : Figure S4d). Overall, the compositions were much more similar within B3 than those between B3 and founding workers at the SDP level (Fig. 1f).

**Fig. 2:**
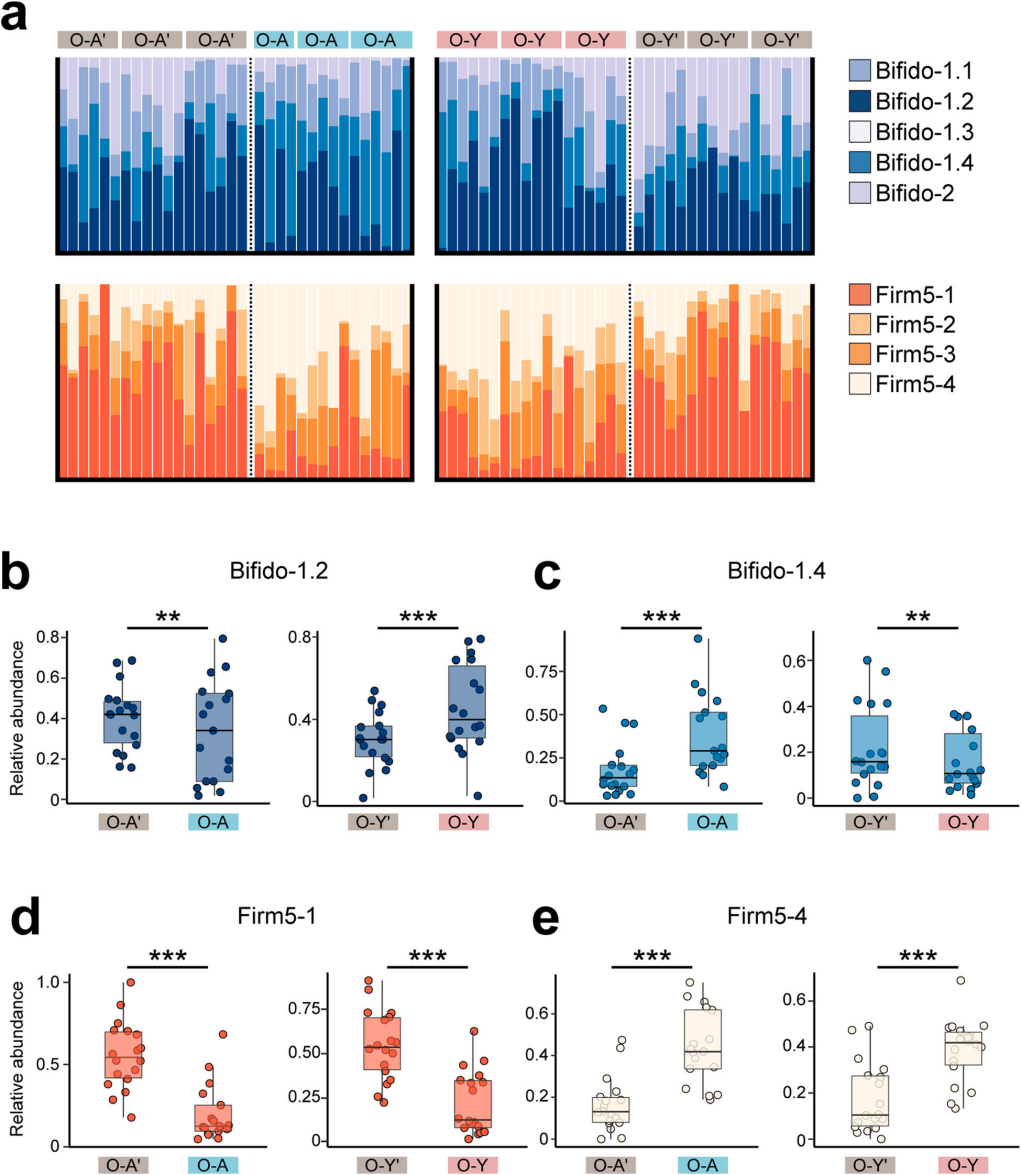
Relative abundance of bacterial species varies between hybrids and founding bees. **a** Relative abundance profiles of SDPs from *Bifidobacterium* and *Lactobacillus* Firm5 for the founding workers (O-A’, O-Y’) and the B3 batch of individuals (O-A, O-Y). **b–e** Comparison of the relative abundances of the SDPs of *Bifidobacterium* Bifido-1.2 (**b**) Bifido-1.4 (**c**), *Lactobacillus* Firm5-1 (**d**), and Firm5-4 (**e**) between the founding workers and the B3 individuals. Wilcoxon test was used to compare the relative abundance of each microbiota taxon between each two bee groups. (**p < 0.01; ***p < 0.001)

While the relative abundances of most SDPs were not significantly different, Bifido-1.2 and Bifido-1.4 were differentially distributed in the genetically varied hosts (Fig. 2a). Interestingly, Bifido-1.2 was enriched in the O-A’ founding workers, as compared to O-A. However, it was more abundant in the B3 of O-Y bees (Fig. 2b). By contrast, Bifido-1.4 was more abundant in O-A but decreased in O-Y than the founding workers (Fig. 2c). These strongly suggested that the host genetics showed different selection powers upon the bacterial SDPs. For *Lactobacillus* Firm5, all founding workers had a higher fraction of the Firm5-1 cluster, whereas the B3 bees harbored more Firm5-4 in the gut (Fig. 2d, e). Altogether, our results indicated that some SDPs shifted in relative abundances in hosts with differential genetic backgrounds during transmission, and the host genotype could shape the pattern of gut microbiota transmission within the colony.

### Biased SNP distribution in Type IV pili (T4P) structural component-coding genes underlying the strain-level difference in *Snodgrassella*

**Fig. 3:**
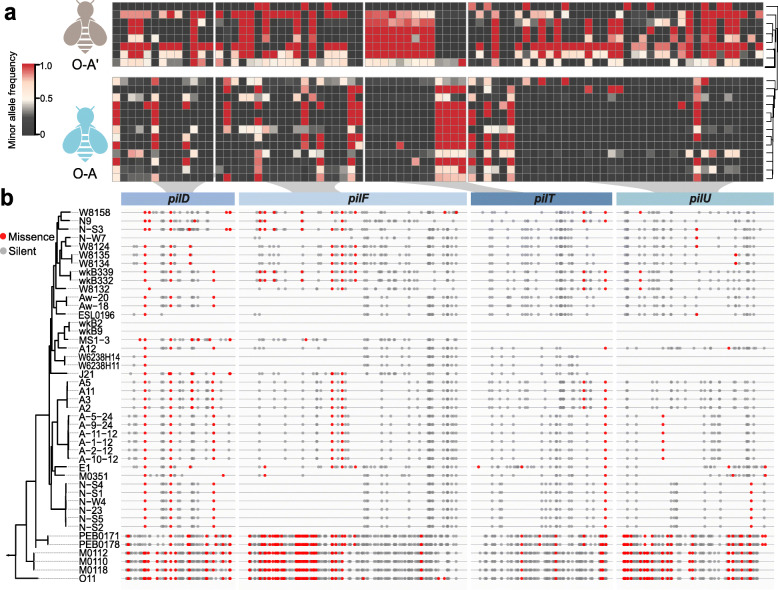
SNPs of Type IV pili component genes of *Snodgrassella alvi* are differentially distributed between the founding workers and the B3 bees in the colony. **a** A heatmap showing the minor allele frequency for missense SNPs is significantly different in founding workers (O-A’) and the B3 batch of bees (O-A). Each row represents one bee metagenomic sample, and each column is one site in the T4P genes. The tree on the right illustrates a dendrogram of clustering (Ward’s method). **b** Whole-genome phylogenetic tree of isolated *Snodgrassella* strains using the maximum-likelihood algorithm based on the concatenation of core protein sequences. The lines aligned to tree leaves represent corresponding gene sequences with missense (red dots) and silent (grey dot) SNPs

It has been shown that strains of the same SDP from the bee gut are genetically divergent, and the strain-level profiles can be different among individuals from the same colony [19]. Unlike other core members, *Snodgrassella* possess only one SDP, and they specialize in colonizing the hindgut epithelium, suggesting its relatively close interactions with the host [53]. To identify if hosts with different genetic backgrounds have characteristic strain compositions, we analyzed the differences in genome SNP distribution of *Snodgrassella* strains between the founding workers and the B3 individuals. Herein, the distribution of the minor allele frequency per genomic site was used to indicate the strain variations in different hosts. An examination of the SNP distributions of protein-coding genes of *Snodgrassella* revealed 1,547 genes with a valid coverage in 52 bee individuals. Among these genes, 1436 genes possessing sites with significantly differentiated allele frequencies were identified between the two groups of bees (Mann-Whitney test, *p* < 0.05). Notably, four genes encoding the T4P harbored the most differentiated SNP distributions with significant enrichment of group-biased SNPs between O-A and O-A’ bees, while the coverage for the O-Y group was not sufficient for the downstream analysis (Fig. 3a, Additional file : Dataset S2). Firstly, with the reference strain of wkB2, we identified 1,017 SNPs in the *pilD* (prepilin peptidase), *pilF* (fimbrial biogenesis protein), *pilT* (pilus retraction/twitch motility motor), and *pilU* (prepilin peptidase) genes in the genomes of *Snodgrassella* isolates, and there were generally more SNPs (both missense and silent) in phylogenetically distant strains (e.g., strains PEB0171 and M0112; Fig. 3b). The heatmap presented all missense SNP sites with significantly biased distributions between the O-A and O-A’ groups (Mann-Whitney test, *p* < 0.05), and the dendrogram based on SNP frequencies exhibited two different clustering groups, according to the host genotype (Fig. 3a). These indicated that (i) *Snodgrassella* strains exhibited a markedly different enrichment of SNPs in the T4P genes; (ii) a specific set of strains were found to be correlated with genetically varied bee hosts. Genome-wide Tn-seq analysis has documented that the T4P genes of *Snodgrassella* are beneficial for gut colonization and are potential determinants of host specificity [53]. These indicated that the host genotype may have a strong effect on the strain-level composition of gut bacteria and that the T4P of bacteria plays vital roles in microbiota-host interactions [54].

### GWAS revealed that the relative abundance of *Bifidobacterium* is associated with host genetic variants

Since a correlation was observed between the host genotype and the gut composition, the heritability of each taxon was then estimated, and GWAS was performed to identify which host gene was most associated. Both the host genotype and gut community composition data of 102 individuals (including 68 pure bees, 34 hybrid bees) were included in this analysis. The proportions of six core bacterial phylotypes and 17 SDP-level compositions were treated as individual traits, and 2,861,994 informative SNPs spaced throughout the honey bee genome were included (Additional file : Figure S5a). We used both a mixed-model algorithm and an interactive usage of fixed and random effect models to correct the population structure for GWAS. According to the simulation and permutations, the threshold for genome-wide significance was corrected for multiple testing with a weighted Bonferroni adjustment, as previously described [55]. While most of the associations did not reach the study-wide significance, the compositions of the Gilli-2 and Bifido-1.4 SDPs were found to be significantly associated with the host genotype (Additional file : Figure S5a). Heritability analysis revealed that both SDPs showed a relatively higher heritability (Additional file : Figure S5b).

**Fig. 4:**
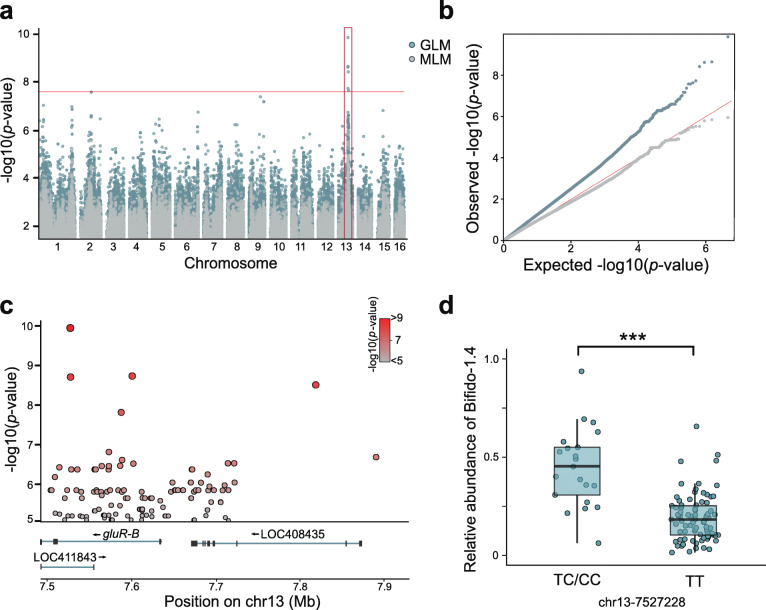
Relative abundance of Bifido-1.4 is associated with host genetic variants. **a** Genome-wide Manhattan plot: each dot represents the -log of the *p* value for the association of the SNP with the relative abundance of Bifido-1.4. The red box highlights the associated locus on chromosome 13 containing the gene *gluR-B*. The threshold for genome-wide significance was corrected for multiple testing with a weighted Bonferroni adjustment. The threshold value was set at -log(*p*) > 2e−8. We used both the General Linear Model (GLM) and Mixed Linear Model (MLM) for the association tests. **b** Quantile-quantile plot showing deviation from the expected distribution of *p* values. The diagonal (red) line represents the expected distribution. **c** Close-up plots of ~ 0.4-Mb window around the SNPs with the highest associations. The coloring of each circle is proportional to the significance. The exon-intron architecture of the *gluR-B* gene and two neighboring genes (LOC408435, LOC422843) are shown at the bottom. **d** Relative abundance of the Bifido-1.4 SDP in bees with different genotypes at the *gluR-B*-associated SNP (chr13-7527228; *p* < 0.001, Wilcoxon test)

GWAS using both GLM and MLM revealed that the SNPs with the strongest association to the relative abundance of the Bifido-1. 4 SDP lies within the *gluR-B* gene located on chromosome 13 (*p* < 0.05; Fig. 4a, b). GluR-B is a metabotropic glutamate receptor (mGluR) that regulates glutamatergic synapses and is specifically expressed in the honey bee brain [56]. Close observation showed that the *gluR-B* gene was enriched by 112 SNPs highly associated with the Bifido-1.4 SDP (Fig. 4c). Correspondingly, bee individuals carrying the TC/CC allele have higher levels of Bifido-1.4 than those carrying the TT allele at the locus with the strongest association (chr13-7527228; Fig. 4d). In addition, the relative abundance of Gilli-2 in the gut is correlated with the SNPs from multiple genes encoding inositol-pentakisphospate 2 kinase, carboxypeptidase Q, and poly(rC)-binding protein within chromosome 12 (Additional file : Figure S5a).

### *Bifidobacterium* alters alternative splicing of *gluR-B* and elevates the GABA level in the brain

**Fig. 5:**
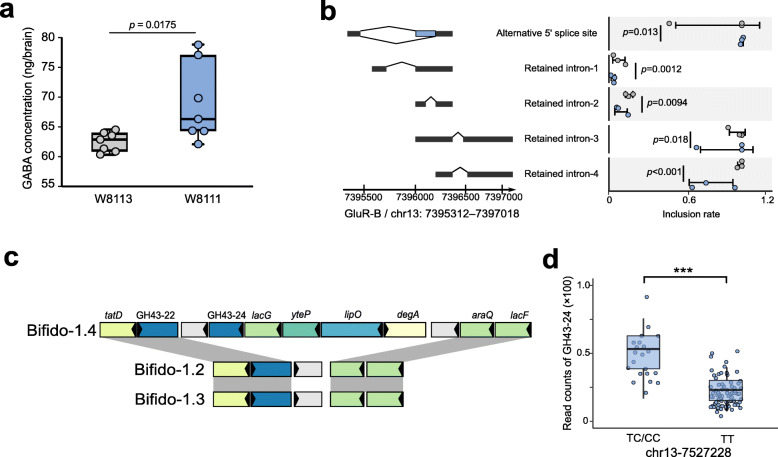
Bifido-1.4 with a unique PUL-like gene cluster impacts gene expression and GABA concentration in the brain. **a** Targeted metabolomics indicates that the GABA concentrations are increased in the brain of Bifido-1.4 (W8111) mono-inoculated honey bees than bees associated with Bifido-1.2 (W8113). **b** Differential splicing events of the *gluR-B* gene in brains of Bifido-1.2 and Bifido-1.4 inoculated bees. Benjamini-Hochberg corrected *p* values are shown. **c** Syntenic loci of the PUL in *Bifidobacterium* strains from different SDPs. Homologous genes are connected by gray bars. **d** Boxplots of the read count for GH43-24 specific to Bifido-1.4 in each genotype at the *gluR-B*-associated SNP (chr13-7527228)

The honey bee mGluR is a G-protein-coupled receptor (GPCR), which shares sequence similarity with the B-type gamma-aminobutyric acid (GABA_B_) receptors, the calcium-sensing receptors, and some pheromone receptors [57]. The mGluR and GABA receptors mutually modulate signal transduction, and its expression is controlled by both glutamate and GABA in the brain [58]. GABA is an inhibitory neurotransmitter found at high concentrations in the honey bee brain, which has been implicated in several honey bee behaviors, including odor coding, learning, and memory, as well as locomotion control [59, 60]. The altered gut and hemolymph metabolome have been associated with the honey bee microbiome; specifically, glutamic acid is enriched both in the gut and the hemolymph of bees with a normal gut community [24]. Since the significant association between GluR-B and the level of gut *Bifidobacterium* were identified, whether the concentration of honey bee brain GABA is affected by the colonization of Bifido-1.4 was next explored. To control the effect of bacteria colonization, we colonized MF bees with isolates W8111 from Bifido-1.4 and another strain W8113 from Bifido-1.2 as the negative control (Additional file : Figure S6). Targeted metabolomics revealed that the GABA concentration in brains of bees mono-colonized (MC) with Bifido-1.4 was significantly higher than that of Bifido-1.2 colonized bees (Fig. 5a).

Given the evidence that *gluR-B* is preferentially expressed in the brains, we investigated whether the colonization of Bifido-1.4 altered the gene expression profiles. Although the expression level was not changed (data not shown), RNA sequencing analysis revealed that *gluR-B* exhibited differential patterns of AS events between MC bees associated with strains of *Bifidobacterium* (Fig. 5b). Bees colonized with strain W8111 showed decreased inclusion rates of 5’ alternative start site and four different retained intron events. These results suggested that the glutamate receptor might be regulated by GABA in the brain [61]. Moreover, the colonization of specific gut strains affects AS of brain genes, which regulates the production of isoforms of the mGluR gene.

Since strains from different SDPs of *Bifidobacterium* varied in their ability to alter host brain physiology, we sought to identify gene presence/absence signatures that may explain the inheritance patterns of different *Bifidobacterium* SDPs. We wonder why strains of the Bifido-1.4 SDP are preferentially transmitted and hypothesized that these strains possess specific genes implicated in their interactions with the host. It has been documented that *Bifidobacterium* are the major degraders of diet hemicellulose, and the abilities of individual strains vary [34]. Therefore, we searched for genes present in Bifido-1.4 but absent in the other SDPs of *Bifidobacterium*. A total of seven genes that are specific to Bifido-1.4 strains were identified, and they were located in a single genomic region of the reference genomes (Fig. 5c). This region contained two carbohydrate-active enzymes (CAZyme) of GH43-22 and *lacG* encoding phospho-beta-galactosidase, the LacI-family transcriptional regulator *araQ* helix-turn-helix type transcriptional regulator *degA,* and a multiple-sugar transport system permease *yteP*. They form a typical structure of the CAZyme gene cluster, enabling them to specialize in the breakdown of dietary fiber. To confirm if host genetics are associated with the functional capacity of the gut bacteria, the read counts of the GH43-22 gene were compared in bees showing different alleles (Fig. 5d). Our metagenomic analysis revealed an enrichment of the glycoside hydrolase GH43-22 in the guts of individuals with TC/CC at the locus chr13-7527228, suggesting that the bee genotype is associated with an abundance in polysaccharide-degrading gut microbes.

## Discussion

In the present study, the socially transmitted gut microbiota of purebred and hybrid honey bees were used to elucidate the consequences of host genetic divergence in the composition of symbionts. Our metagenomic analysis characterizing the microbial structure at different taxonomic levels represents strong evidence that specific gut members are affected by the host genetic factor during the transmission. Moreover, bee individuals were genotyped simultaneously with the characterization of the microbiome, allowing for tests of the association between host SNPs and microbiome traits. Finally, we focused on the heritable gut member, *Bifidobacterium* sp. Bifido-1.4 and its colonization altered the brain neurotransmitter and gene expression patterns, which might be associated with a unique PUL-like region in the genome.

It has been well documented that the honey bee gut microbiota are dominated by limited numbers of bacterial phylotypes, commonly with species from the *Gilliamella*, *Snodgrassella*, *Lactobacillus*, *Bifidobacterium,* and *Bartonella* genera. Therefore, it is not surprising that the variation was not obvious among individuals at the phylotype level (Fig. 1). Although the core gut members have been observed in bees across studies, even in samples from different countries, gut microbiota can differ markedly in diversity across adult bees [17]. Several factors are influential in the composition of the bee gut community, such as the regional floral diversity, seasonal shift, age, and bee caste [27, 62, 63]. Nevertheless, the environmental factors may contribute to diet preference and nutritional status, which are the main drivers of community variance. However, certain studies have also reported an intercolonial difference among individuals belonging to the same caste and sampled at the same age. Specifically, high levels of strain-level diversity exist within all major phylotypes in the honey bee gut. By targeting protein-coding gene markers of *Gilliamella* and *Snodgrassella*, it was found that strain compositions of individuals from the same hive can vary dramatically, and some strains are specific to only one host species [22, 64]. Such strain-level diversity was also recently appreciated by community-wide shotgun sequencing [20]. Since all studies have focused on colonies naturally headed by hyperpolyandrous queens, we hypothesized that both environmental factors and host genetics shaped the diversity in the bee gut microbiota. Our findings clearly showed that the abundance in both phylotypes and SDPs was more correlated within bees from the same subspecies, indicating the host genetic interactions with specific gut members. In humans, comparative analyses revealed that family members have more similar microbiota, partly due to shared environmental influences [65, 66]. Although we sampled bees from outdoor hives, we controlled individual age and sampled simultaneously from multiple colonies reared at the same apiary to minimize factors other than host genetics. While identical environmental conditions cannot be guaranteed for these colonies, and the pollen/nectar sources might change according to the progress of season (e.g., possibly a bias for flower preference), no bias in the flower preference of honey bees was identified in the host genetics [67].

In bee colonies, the gut symbiotic bacteria are transmitted between generations of siblings through social contact. Thus, the core lineages of gut bacteria show phylogenies matching those of the hosts, highlighting codiversification along with the evolutionary history of symbiosis. Mounting evidence has demonstrated codiversification via a long-term vertical association in many animal groups, and host filtering could be a major driver of “phylosymbiosis” [1]. A considerable shift in community composition was found during the transmission in our microbiota passaging approach in two independent lines of hybrid bee populations. Notably, the highest divergence was observed between the founding workers and B1, indicating that host genotype shapes the gut composition early in passaging. Although it was found that all SDPs from the core phylotypes can be inherited by the hybrid bees during the consecutive transmission within the hive, the effect of the host differed widely on even closely associated discrete bacterial populations from the same phylotype. For two SPDs from *Lactobacillus* Firm5, the host filtering processes were similar in O-A and O-Y hybrids. By contrast, the relative abundance of *Bifidobacterium* Bifido-1.2 was decreased in O-A but increased in O-Y bees (Fig. 2). This shows that genetically varied hosts permit the colonization of specific sets of bacteria, strongly suggesting that the effect of the genotype is driving the microbiota structure.

While we observed a noticeable shift in the gut community between the founding bees and the first batch of O-A, the microbiota was not significantly altered during transmission in O-Y (Fig. 1e). This suggests that the effects of genetic variance among hosts could be different. Interestingly, the divergence of host genetics in the O-A group was lower than that among O-Y hybrids (Additional file : Figure S4c), which might partly explain the observed filtering process. When the living environments and microbial populations are controlled, the influences of host genetics on microbial compositions have also been detected in other animals and plants [68, 69]. However, the composition of resultant output microbiota is always determined by the input species [70]. In our transfer experiment, the output microbiomes were compared within two parallel lines with a nearly identical starting community carried by the same batch of founding workers; however, they were passaged in genetically different hosts, allowing us to overcome the legacy effects.

So far, only one SDP from *Snodgrassella alvi* was identified, but a markedly high level of strain diversity was observed based on the proportion of polymorphic sites in core gene sequences [19]. For bees from the same colony, it was found that strains can be dominant in one individual and absent in another [64]. Although the GWAS associated host genetic loci to the overall microbiome divergence (beta-diversity) or the abundances of several taxa, few studies have focused on the strain composition, which is primarily due to the markedly high level of diversity at this level. The presence of only one lineage of *Snodgrassella* with genetically divergent strains enables the fine-scale analysis of the effect of the host on strain stability. It was found herein that strains are subject to the selection of host genotype, and different strains are enriched in genetically varied hosts during the social transmission. This suggests that bees with different genetics have an elaborate mechanism to ensure specificity of the association, which might be achieved by signal recognition and secretion of antibacterial agents, as found in the symbioses of microbes and both plants and animals [71, 72]. Interestingly, it was found that the distribution of SNPs in T4P genes was significantly biased between genetically different hosts. T4P encodes membrane-associated transporter complex, which is essential for biofilm formation and cell adhesion and has been identified as significant for the survival of pathogenic bacteria in eukaryotic hosts [73]. *Snodgrassella* forms a layer attached to the inner gut wall, which is vital for maintaining the gut microenvironment [24]. *Snodgrassella* possesses all core components of T4P in the genome, which facilitate colonization in vivo [53]. Specifically, the ability of surface biofilm formation of structural mutant *pilF*^*−*^ was significantly reduced, and the SNP distribution of *pilF* was found to be affected by the host genotype. Another major pilin subunit, *pilE*, which is beneficial for colonization, is exclusive to strains from the honey bee, suggesting that T4P is a decisive factor in host adaptation. T4P are multicomponent transporters for transferring proteins and DNA into target cells and are critical for the host specificity of most bacterial pathogens by mediating the adhesion and invasion processes [74, 75]. Our findings illustrated that T4P might provide selective advantages for different strains during the transmission in hosts with various genotypes, specifically for *Snodgrassella* colonizing the gut epithelium.

In our dataset, the Bifido-1.4 SDP from *Bifidobacterium* exhibited the most significant association with the host *gluR-B* gene locus and is also a highly heritable taxon. *Bifidobacterium* is also heritable in the human TwinsUK population, the HMP, and mouse models [7, 76–78], implying that *Bifidobacterium* are a group of symbionts critical for the physiology or metabolism of a variety of animal hosts. Indeed, it has been documented that *Bifidobacterium* are the principal polysaccharide degraders for bees, and not all members of SDPs are capable of digesting hemicellulose in diet pollen. Here, Bifido-1.4, with a strong signal in the GWAS, is capable of degrading hemicellulose in vivo and possesses an abundant repertoire of carbohydrate-active enzymes [34], highlighting that the diet interaction is fundamental for the host-gut microbe association. Indeed, in human intestines, the correlation between *Bifidobacterium* and SNPs near the *LCT* gene associated with lactase nonpersistence has been replicated across several populations [11]. This is due to the presence of lactose consumed by lactase nonpersister in the large intestine, which can stimulate the proliferation of *Bifidobacterium* [5]. While the specific strains of *Bifidobacterium* that are implicated in the association with the host genotype of lactose nonpersister were not determined in humans, our analysis specifically illustrated an association between host genetics and strains with more abundant glycoside hydrolase genes (GHs) that assist polysaccharide digestion in the bee gut. To characterize the functional significance of strain inheritance events, we examined genes only present in strains of Bifido-1.4 and not in other SDPs of *Bifidobacterium*. Bifido-1.2 and Bifido-1.4 both enrich GHs forming PUL-like regions in the genome, yet only one is different between these two SDPs, which might provide a selective advantage in colonization [79].

The sites significantly associated with Bifido-1.4 are located in *gluR-B*, which is a member of GPC mGluRs. Two subtypes of mGluRs have been characterized in honey bees, with only *gluR-B* expressed at high levels in the Kenyon cells within the mushroom body [56]. While they are both glutamate receptors, their overlapping expression in the brain suggests that they interact to modulate the glutamatergic neurotransmission and respond to different GABA levels [80], one of the most abundant neurotransmitters in adult bee brains [81, 82]. Although the host genotype has not been linked to the GABA concentration in the brain, altered expression profiles of *gluR-B* were observed with a higher level of GABA in the bee brain (Fig. 5). Taken together, these results point to an interaction between the *Bifidobacterium* and host brain physiology. The causality of the association between the relative abundance of Bifido-1.4 and the host brain neurotransmitter was tested experimentally by gnotobiotic bees. Mono-associated bees with a specific strain of Bifido-1.4 showed an increased level of GABA in the brain as compared to those colonized with Bifido-1.2. Moreover, AS events of *gluR-B* are affected by the gut microbe, which can regulate the production of specific isoforms of genes implicated in host phenotypes. Interestingly, a recent study has linked the human autism spectrum disorder to the microbial metabolism of 5-aminovaleric acid, a weak GABA_A_ receptor agonist [83], which is one of the most elevated metabolites in gnotobiotic bees with conventional microbiota [24]. These suggest that honey bee gut microbiota may regulate host phenotypes via microbial metabolism, implying a gut-brain connection that contributes to impaired behaviors that share common molecular mechanisms with those of humans [84, 85].

Honey bees, as effective pollinators, are instrumental in the production of foods all over the world. A recent decline in the honey bee population has been a major threat to the balance of the global ecosystem. It has been shown that host genetic variation drives the honey bee phenotype, particularly the host nutritional status, colony health, and productivity [86–88]. Our data illustrated that the host genotype influences the socially transmitted gut microbiota assembled at emergence. Given the evidence that bee gut microbiota are largely involved in host health, the present study underscored that honey bee genes might influence health directly or by developing a beneficial microbiota. Honey bees have long been regarded as a model organism for biology studies, such as social behavior, recognition, genetics, and host-microbiota symbiosis [23, 89, 90]. Further identification of host alleles as shaping forces of microbial structure will advance our understanding of the host-microbe interactions.

## Conclusions

Gut microbiota affect host health and can be regulated by host genetics. We show that the gut structures are different among genetically varied bee subspecies, and the compositions dramatically shift during the social transmission among founding workers and hybrid bees. Host genotype has a strong effect on the strain-level composition of *Snodgrassella*, and the bacterial Type IV pili may play an important role in microbiota-host interactions. Furthermore, we identified that the host *gluR-B* gene is associated with the abundance of *Bifidobacterium*. Mono-colonization of *Bifidobacterium* strains with a specific gene suite for polysaccharide degradation can modulate the GABA level and alternative splicing of the host genes in the brain. This work highlights the mechanisms implicated in the host-microbiota cross-talk for honey bees.

## Supplementary Information

**Additional file 1: Table S1. Figure S1-S6.**

**Additional file 2: Dataset S1.** The list and pairwise ANI values of genomes of bacterial isolates in the database for MIDAS profiling.

**Additional file 3: Dataset S2.** Metadata of SNP distribution in Type IV pili component genes of *Snodgrassella alvi* in hybrid bees and founding workers.

## Data Availability

Sequencing data of the metagenomes have been deposited under BioProject PRJNA645015. The genomes of the bacterial isolates have been deposited under BioProject PRJNA648267. Accession numbers for genomes included in the MIDAS genomic database are provided in Dataset S1. Raw data of 16S rRNA-based amplicon sequencing has been deposited under BioProject PRJNA645267. RNA sequencing data of the honey bee brains have been deposited under BioProject PRJNA735887. The list of analysis software and scripts generated for analysis have been deposited on GitHub at: https://github.com/JiaqiangWu/bee_micro.git.

## Abbreviations

GWAS: Genome-wide association study
SDP: Sequence-discrete population
MF: Microbiota-free
SNP: Single-nucleotide polymorphism
T4P: Type IV pili
GPCR: G-protein-coupled receptor
GABA_B_: B-type gamma-aminobutyric acid
MC: Mono-colonized
AS: Alternative splicing
CAZyme: Carbohydrate-active enzymes
GHs: Glycoside hydrolase genes
MIDAS: Metagenomic Intra-Species Diversity Analysis System
GLM: General Linear Model
MLM: Mixed Linear Model
PUL: Polysaccharide utilization loci
