# Supplementary material for: Honey bee genetics shape the strain-level structure of gut microbiota in social transmission
Source: Microbiome. 2021 Nov 17;9:225. doi: 10.1186/s40168-021-01174-y (PMC8597283; doi:10.1186/s40168-021-01174-y)
Supplement: Supplementary file 2 — Additional file 1: Table S1. Figure S1-S6. [file 40168_2021_1174_MOESM2_ESM.pdf]

Supplementary Information

Honeybee genetics shape the strain-level structure of gut microbiota in social transmission

Jiaqiang Wu[1,2], Haoyu Lang[1,2], Xiaohuan Mu[1], Zijing Zhang[1], Qinzhi Su[1], Xiaosong Hu[1], Hao Zheng[1,*]

[1]College of Food Science and Nutritional Engineering, China Agricultural University, 100083 Beijing, China

[2]These authors contributed equally.

*Correspondence: hao.zheng@cau.edu.cn

Table S1 Sample sizes of individual honey bees used for microbiome analysis.

Sample	Sequencing strategy		Total
	16S rRNA	Metagenomic	
**OH group**			
OH		29	29
**AF group**			
AF		8	8
**YF group**			
YF-Hive 1		5	5
YF-Hive 2		5	5
**SK group**			
SK-Hive 1		5	5
SK-Hive 2		5	5
**O-A group**			
O-A-Hive 1			
batch 1	10		10
batch 2	10		10
batch 3	5	4	9
O-A-Hive 2			
batch 1	10		10
batch 2	10		10
batch 3	5	5	10
O-A-Hive 3			
batch 1	10		10
batch 2	10		10
batch 3	5	6	11
**O-A' group**			
O-A-Hive 1	10	6	16
O-A-Hive 2	10	6	16
O-A-Hive 3	10	6	16
**O-Y group**			
O-Y-Hive 1			
batch 1	10		10
batch 2	10		10
batch 3	5	6	11
O-Y-Hive 2			
batch 1	10		10
batch 2	10		10
batch 3	5	6	11
O-Y-Hive 3			
batch 1	10		10
batch 2	10		10
batch 3	5	6	11
**O-Y' group**			
O-Y-Hive 1	10	5	15
O-Y-Hive 2	10	6	16
O-Y-Hive 3	10	6	16
**Total**	**210**	**125**	**335**

*Each row represents one colony, and hybrid bees in different batches were counted separately.

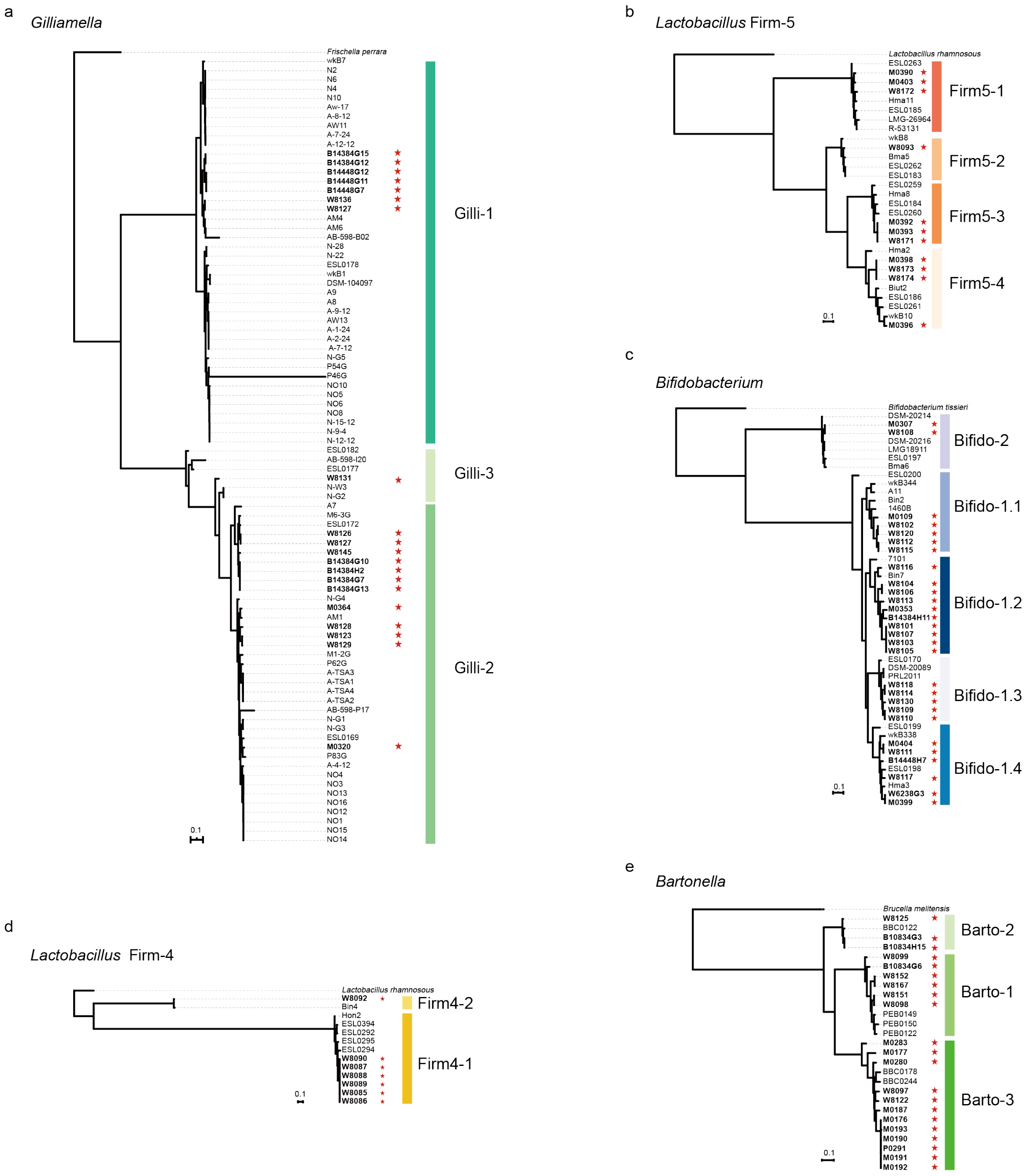

Figure S1. Genome phylogenies and SDP validation of the core bacterial phylotypes and *Bartonella apis* isolated from *Apis mellifera* guts. Phylogenies inferred for *Gilliamella* (**a**), *Lactobacillus* Firm-5 (**b**), *Bifidobacterium* (**c**), *Lactobacillus* Firm-4 (**d**), and *Bartonella* (**e**) based on the genomes of isolates in the reference database using PhyloPhlAn. Newly isolated strains in this study are annotated by a red pentagram.

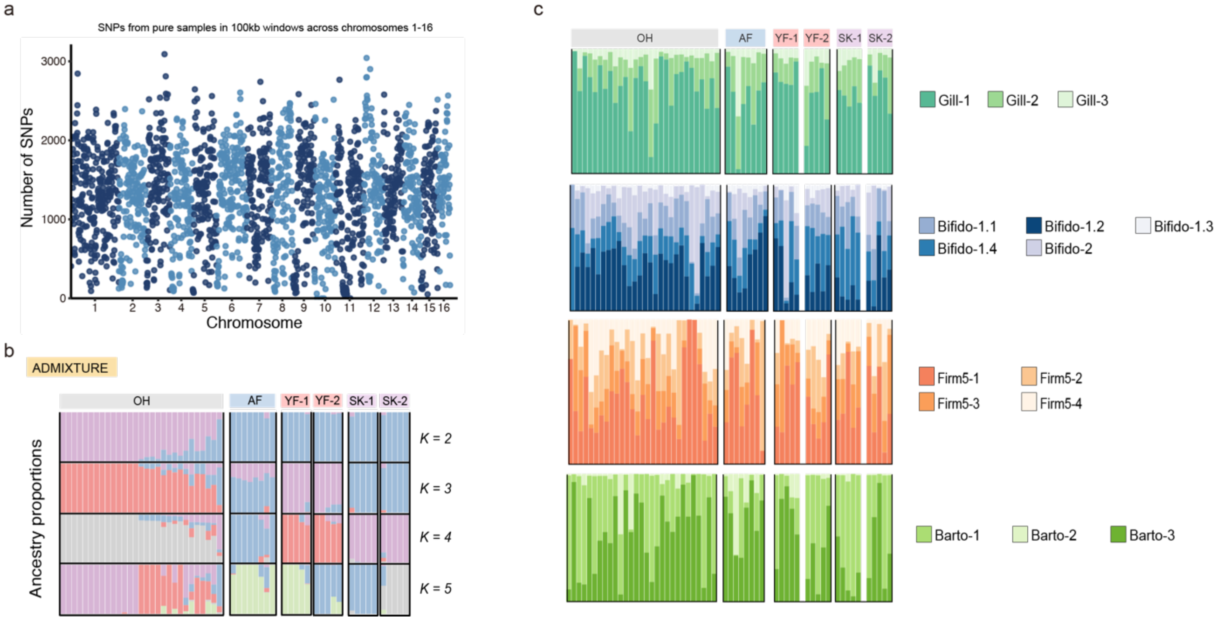

Figure S2. Gut community composition of genetically different purebred honeybees at both phylotype- and SDP-level. (**a**) The number of SNPs from all purebred samples across *Apis mellifera* chromosomes 1 to 16 in 100 kb consecutive windows. (**b**) ADMIXTURE analysis estimates the proportion of each bee genome derived from each of assumed populations (K = 2–5). The inferred proportion of ancestry is shown for each sample. (**c**) Bar plots showing the relative abundance of the core phylotypes and the SDPs from each phylotype in each subspecies group.

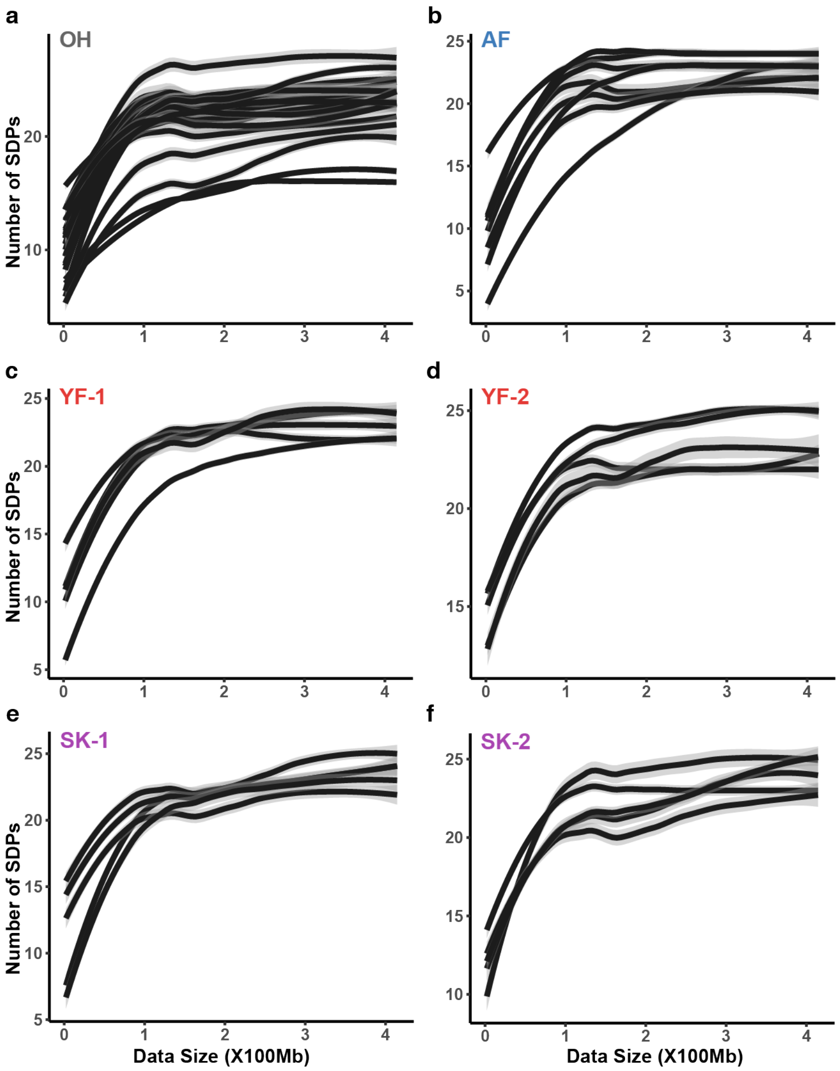

Figure S3. Cumulative number of observed SDPs for each metagenomic sample. The calculation of SDP richness for a given data size for each individual bee. Each panel represents one colony of a purebred subspecies, each line represents one bee individual, and the shaded area represents the 40% confidence intervals.

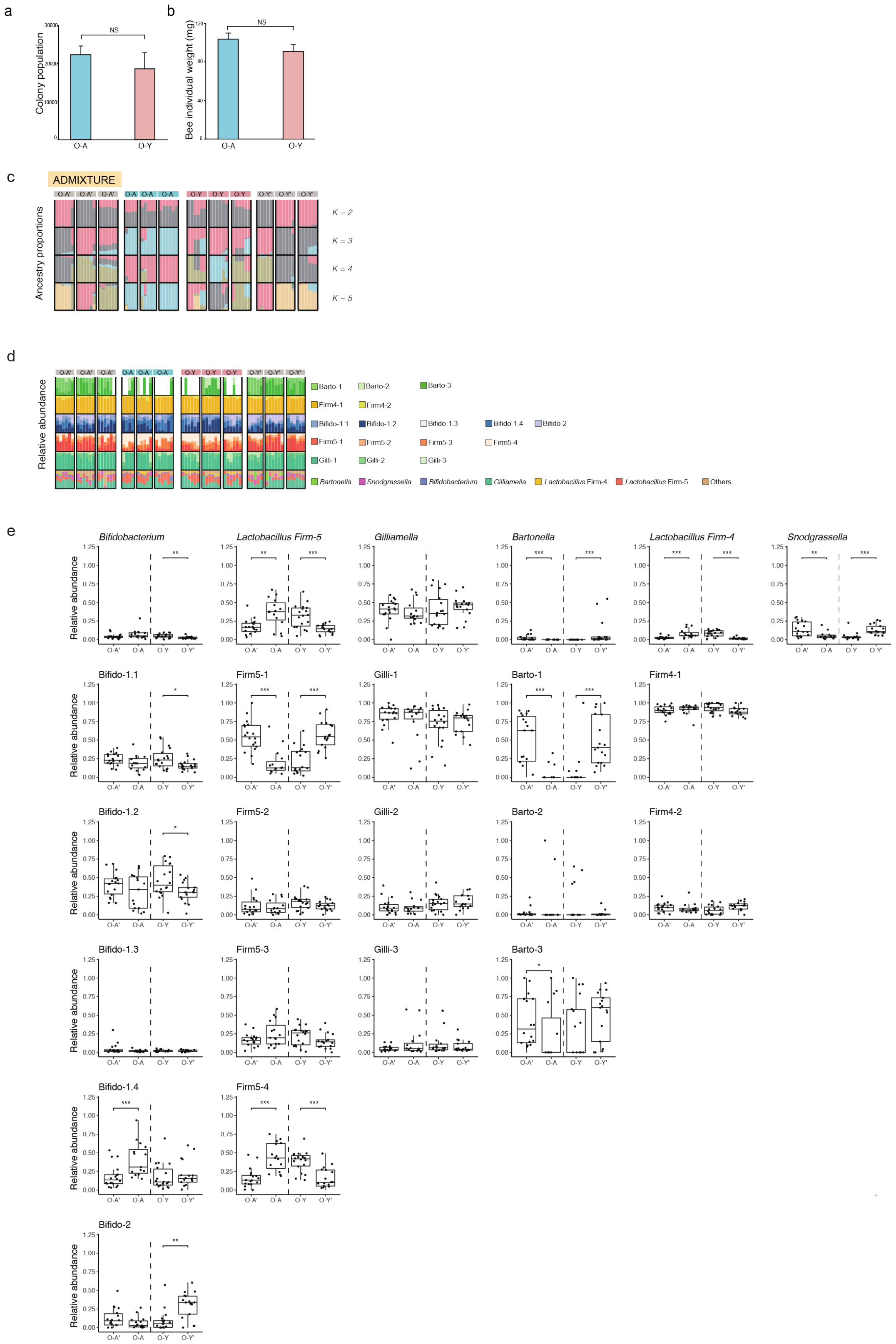

Figure S4. Gut community composition of founding workers and hybrid honeybees at both phylotype- and SDP-level. There were no significant differences in population size (**a**) or average bee weight (**b**) between the two groups of hybrid bees. (**c**) ADMIXTURE analysis showing clustering of samples into 2–5 groups (*K* = 2–5). (**d**) The inferred proportion of ancestry shared with each group is shown for each sample. (**e**) Boxplots showing the relative abundance of the core phylotypes and the SDPs from each phylotype in each subspecies group. The significance of difference among the four groups and between each pair of groups were texted (*, $p < 0.05$; **, $p < 0.01$; Wilcoxon test).

a

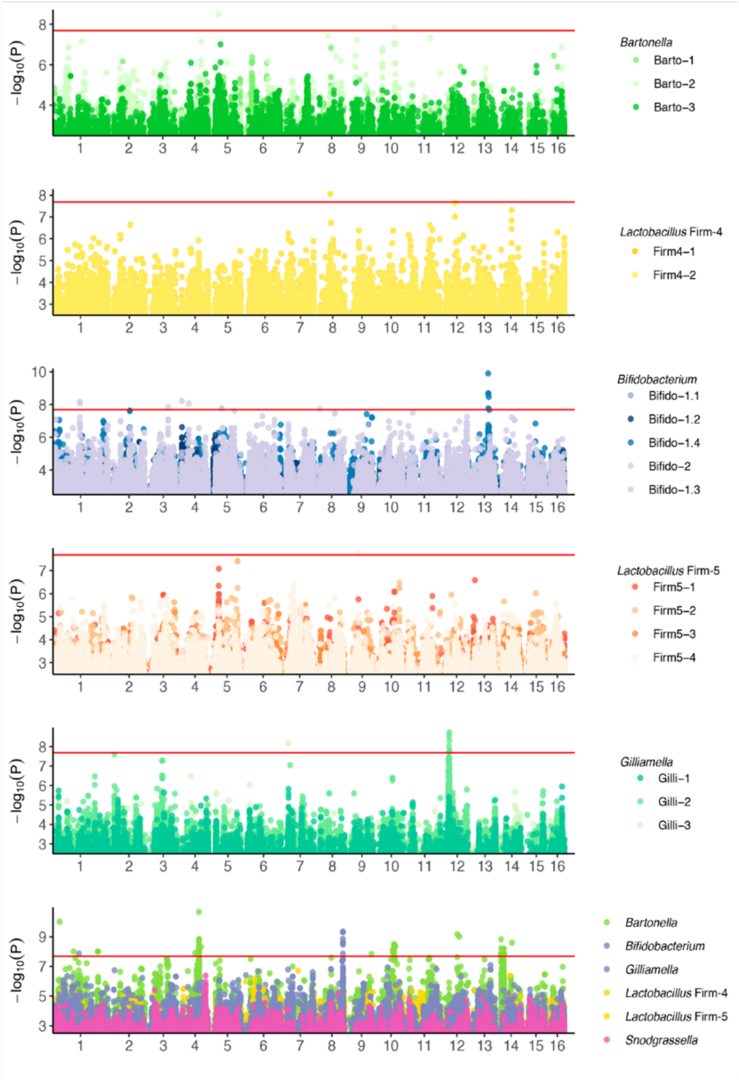

b

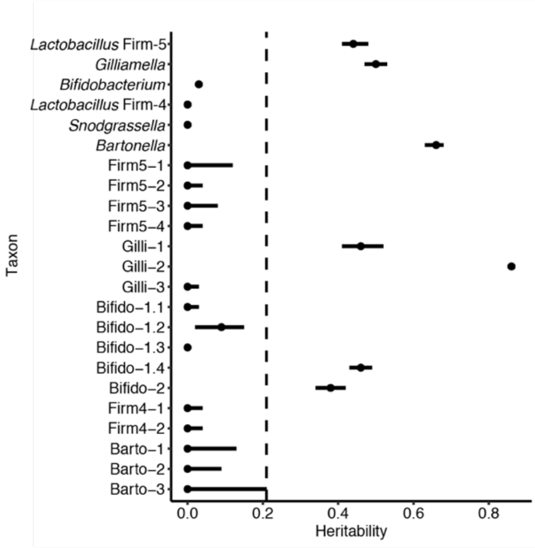

Figure S5. Genome-wide association analysis and heritability estimation for different phylotypes and SDPs. (**a**) Manhattan plot of genome-wide associations with different phylotypes and SDPs. The red line corresponds to a significance threshold of 2×10^{-8}. (**b**) Heritability estimation for phylotypes and SDPs. Each point represents the heritability estimated as the proportion of variance in the relative abundances. The bars show the 95% confidence intervals around the heritability estimates.

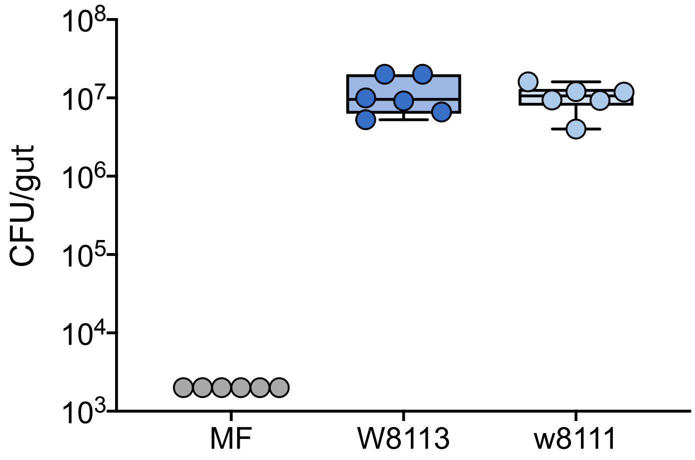

Figure S6. Box plots showing the total CFU per gut estimated by bacteria culture for MF honey bees and bees mono-colonized with Bifidobacterium asteroides stain W8111 and W8113.
